# Effect of the LPA-mediated CXCL12-CXCR4 axis in the tumor proliferation, migration and invasion of ovarian cancer cell lines

**DOI:** 10.3892/ol.2014.1926

**Published:** 2014-02-28

**Authors:** HUI WANG, WENLI LIU, DONGMIN WEI, KUN HU, XIAOHUA WU, YUANQING YAO

**Affiliations:** 1 Department of Obstetrics and Gynaecology, General Hospital of PLA, Beijing 100853, P.R. China; 2 Department of Obstetrics and Gynaecology, Shijingshan Teaching Hospital of Capital Medical University, Beijing Shijingshan Hospital, Beijing 100043, P.R. China; 3 Affiliated Hospital of Hebei University of Engineering, Handan, Hebei 056002, P.R. China; 4 Department of Obstetrics and Gynaecology, Bethune International Peace Hospital of PLA, Shijiazhuang, Hebei 050000, P.R. China

**Keywords:** ovarian neoplasm, lysophosphatidic acid, CXCL12-CXCR4 axis, metastasis

## Abstract

Ovarian cancer is the most fatal gynecological cancer, with a 5-year survival rate of only 30%. Lysophosphatidic acid (LPA), which possesses growth factor-like functions, is a major regulatory factor in the peritoneal metastasis of ovarian cancer. LPA stimulates the expression of numerous genes that are associated with angiogenesis and metastasis. Ovarian epithelial carcinoma specifically expresses chemotactic factor C-X-C motif chemokine ligand 12 (CXCL12) and its receptor, CXC receptor 4 (CXCR4). The CXCL12-CXCR4 axis directly contributes to ovarian cancer cell proliferation, migration and invasion. The present study investigated the regulation of LPA on the CXCL12-CXCR4 axis and the effect of the LPA-mediated CXCL12-CXCR4 axis on the tumor proliferation, migration and invasion of ovarian cancer cell lines. The CXCR4 proteins expressed in the cell membrane and the cytoplasm of ovarian cancer cells, CAOV3 and SKOV3, were detected by immunocytochemistry. The expression of CXCR4 and CXCL12 was increased in the ovarian cancer cells in a dose- and time-dependent manner when treated with LPA compared with the control groups (P<0.05), as determined by reverse transcription polymerase chain reaction and flow cytometry. LPA (20 μM) and CXCL12 (100 ng/ml) enhanced the proliferation, migration and invasion of the ovarian cancer cells, CAOV3 and SKOV3, as identified by MTT, Transwell and Matrigel assays following co-treatment for 24 h. LPA promoted invasiveness of ovarian cancer by upregulating CXCL12-CXCR4 axis expression.

## Introduction

Ovarian cancer possesses unique rules for growth and metastasis; the cancer cells adhere to peritoneal mesothelium cells and form metastases, which then spread widely over the omentum, gastrointestinal tract, liver and spleen, finally leading to patient mortality. A study by Xu *et al* (1) isolated and purified the lysophosphatidic acid (LPA) from the ascites of ovarian cancer patients in 1995 and found a significant increase in LPA levels in the plasma (2). LPA may stimulate growth factors, including vascular endothelial growth factor (VEGF), and interact with the special G protein-coupled receptor on the cell surface (3). LPA may induce cell proliferation, migration and apoptosis, and serve as a tumor marker, indicating that LPA may play significant roles in the occurrence and development of malignant disease. A previous study has shown that LPA could upregulate the protein levels of cyclin D1 in ovarian cancer cells, so as to promote the proliferation of the cancer cells by changing cell progression (3). LPA could also reduce the sensitivity of ovarian cancer cells to cisplatin (4). At the same time, LPA increases the secretion of urokinase plasminogen activator (5) and the expression of VEGF (6) at the transcriptional and protein levels. Additionally, LPA stimulates cell migration, induces new angiogenesis and promotes ovarian cancer growth and metastasis. LPA has also been shown to induce the expression of other genes, including matrix metalloproteinase (MMP) (7), interleukin-8 (8) and cyclooxygenase-2 (9), to promote ovarian cancer cell proliferation and metastasis. This indicates that LPA may promote the invasion and metastasis of ovarian cancer by regulating the gene expression involved in angiogenesis and metastasis.

Certain malignant tumors tend to metastasize to special organs, and the synchronization and specific expression of chemokines and their receptors may play a significant role in the progression of tumors. Recent studies have shown that the C-X-C motif chemokine ligand 12 (CXCL12)-CXC receptor 4 (CXCR4) biological axis, which is composed of chemokine CXCL12 and its specific receptor, CXCR4, plays a significant role in the dissemination of numerous tumors and specific organ metastasis (10–12). In a previous study on ovarian cancer, out of 14 chemokine receptors only CXCR4 was expressed on the ovarian cancer surface, and CXCL12 was detected in the ascites of 63 patients. However, there was no expression of CXCR4 on the surface of the normal ovarian epithelium (10).

Whether LPA can affect the metastasis of ovarian cancer by enhancing chemotaxis, or whether the CXCL12-CXCR4 axis plays roles in the progression of the LPA-promoted peritoneal metastasis of ovarian cancer remains unclear.

## Materials and methods

### Reagent

LPA (1-oleolyl, 18:1) was obtained from Sigma (St. Louis, MO, USA) and dissolved in phosphate-buffered saline (PBS) containing 0.5% fatty acid-free bovine serum albumin (Sigma). The human recombinant, CXCL12 (rhSDF-1), was obtained from Peprotech Inc., (Rocky Hill, NJ, USA), while the mouse anti-human CXCR4 monoclonal antibody and the CXCL12 ELISA kit were obtained from R&D Company (Minneapolis, MN, USA) and Matrigel was obtained from BD Biosciences (no. 354234; San Jose, CA, USA).

### Cell lines and cell culture

The established ovarian carcinoma cell lines, CAOV3 and SKOV3, were generously provided by the Fourth Clinical Medical College Research Center of Hebei Medical University (China). The cells were maintained under standard conditions (37°C and 5% CO_2_) in 75-cm^2^ tissue culture flasks and grown in minimum essential medium supplemented with 10% fetal bovine serum (FBS).

### Immunocytochemical staining

CAOV3 and SKOV3 cells that were grown on coverslips were rinsed in PBS and fixed with 4% paraformaldehyde in PBS for 15 min at room temperature. Immunocytochemical staining was performed as described previously (13), using mouse anti-human CXCR4 monoclonal antibody (1:100).

### Flow cytometry

The CAOV3 and SKOV3 cells were cultured at a concentration of 2×10^5^ cells/ml on 12-well plates and incubated at 37°C. After 24 h, the medium was replaced with serum-free medium. Following an additional 24 h, the serum-free medium was replaced with fresh serum-free media containing various concentrations of LPA. The plates were returned to the incubator for an additional 24 h. A concentration of 1×10^6^/ml single cell suspension was treated with 100 μl rabbit anti-human CXCR4 monoclonal antibody (1:50) and goat anti-rabbit phycoerythrin-conjugated immunoglobulin G, and after 50 min was analyzed by flow cytometry. The relative protein content was indicated by X-mode value.

### Reverse transcription polymerase chain reaction (RT-PCR)

Total cellular RNA was isolated from cultured cells using TRIzol (Invitrogen Life Technologies, Carlsbad, CA, USA). Complementary DNA (cDNA) was synthesized using the High-Capacity cDNA RT kit (Applied Biosystems, Foster City, CA, USA). All PCR reactions were performed under the following conditions: An initial denaturation step at 95°C for 3 min, followed by 33 cycles of denaturation at 94°C for 30 sec, annealing at 56°C for 30 sec and extension at 72°C for 30 sec. The primer sets that were used for the PCR were: GAPDH forward, 5′-TGGTATCGTGGAAGGACTCATGAC-3′ and reverse, 5′-AATGCCAGTGAGCTTCCCGTTCAGC-3′, yielding a 183-bp product; CXCR4 forward, 5′-GAACTTCCTATG CAAGGCAGTCC-3′ and reverse, 5′-CCATGATGTGCTGAA ACTGGAAC-3′, yielding a 304-bp product.

### ELISA

The cell supernatants were collected and then operated in accordance with the ELISA kit instructions.

### 3-(4,5-dimethylthiazol-2-yl)-2,5-diphenyltetrazolium bromide (MTT) assay

For the quantitative proliferation assays, the ovarian cancer cells, SKOV3 and CAOV3, were seeded onto 96-well plates (5×10^3^ cells/well) for 24 h and then the culture medium was replaced with the following media: i) Serum-free RPMI 1640 medium; ii) serum-free RPMI 1640 medium + 20 μM LPA; iii) serum-free RPMI 1640 medium + 100 ng/ml CXCL12; iv) serum-free RPMI 1640 medium + 20 μM LPA + 100 ng/ml CXCL12; and v) serum-free RPMI 1640 medium + 20 μM LPA + 100 ng/ml CXCL12 + 100 ng/ml pertussis toxin (PTX). The cells were treated with MTT (Sigma) after 24 h, and then the absorbance was detected (λ_experiment_=490 nm and λ_control_=620 nm) by enzyme-linked immune detector. The protocol was as described previously (13). The proliferation rate was calculated as: [(λ_experiment_ − λ_control_)/(λ_control_ − λ_blank_) × 100].

### Transwell migration assay

Cell migration was monitored using a Transwell chamber assay. The SKOV3 and CAOV3 cells (2×10^5^ cells) were plated on 8-μm Transwell filters (Corning Inc., Corning, NY, USA). The cells were induced with medium to migrate towards medium without FBS for 20 h (5,6) as follows: i) Serum-free medium; ii) serum-free medium + 20 μM LPA; iii) serum-free medium + 100 ng/ml CXCL12; iv) serum-free medium + 20 μM LPA + 100 ng/ml CXCL12; and v) serum-free medium + 20 μM LPA + 100 ng/ml CXCL12 + 100 ng/ml PTX. Non-migrating cells were removed with a cotton swab. The remaining cells were fixed, stained with hematoxylin and eosin (HE) and analyzed using a bright-field microscope (ECLIPSE Ti-S, Nikon, Tokyo, Japan).

### Invasion assay

The upper chambers of the Transwell filters (Coring Inc.) were coated with 100 μl Matrigel and subsequently SKOV3 and CAOV3 cells (2×10^5^ cells). The medium in the lower chambers was the same as that indicated for the Transwell migration assay. The protocol used was described previously (13).

### Statistics

The statistical analysis was completed with SPSS 11.0 (SPSS, Inc., Chicago, IL, USA). Statistical comparisons were analyzed by t-test, one-way analysis of variance and Student-Newman-Keuls test. Statistical data were indicated by comparing mean ± standard deviation values, and P<0.05 was considered to indicate a statistically significant difference.

## Results

### Expression of CXCR4 in ovarian cancer cells

**Figure 1:**
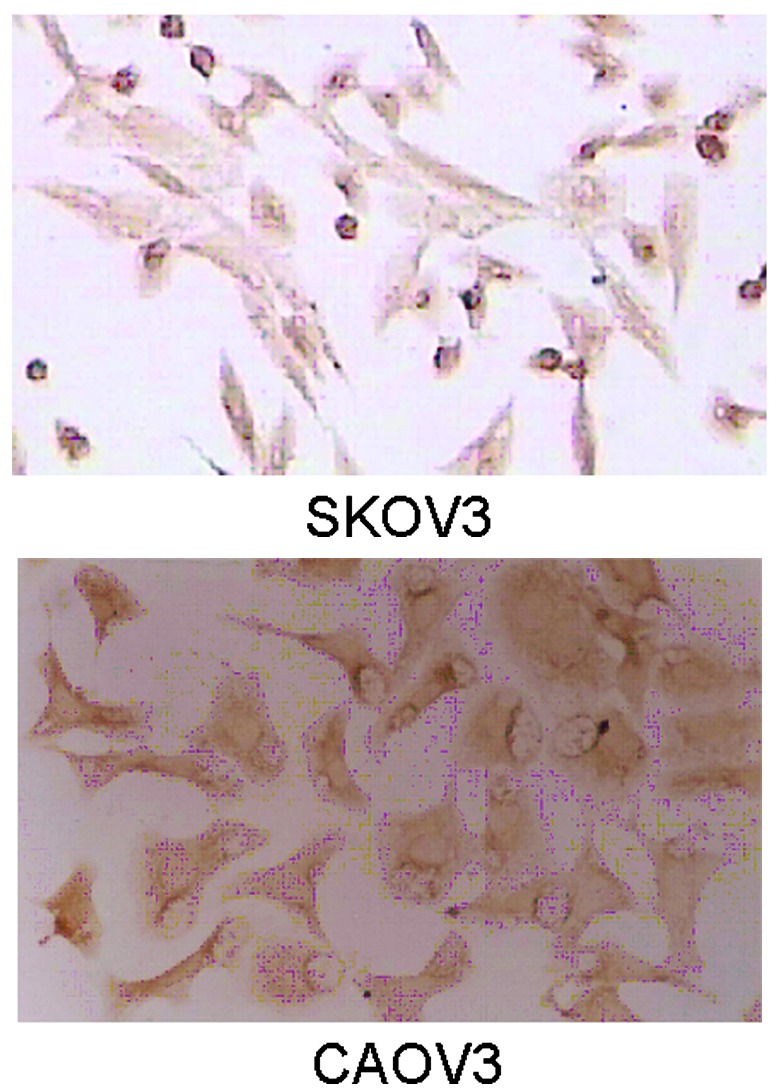
CXCR4 protein expression in SKOV3 and CAOV3 cell lines, as determined by immunocytochemistry (streptavidin-perosidase staining; magnification, ×400). CXCR4, C-X-C motif chemokine receptor 4.

Immunocytochemistry results showed that the CXCR4 protein was positively expressed in the cell membrane, cytoplasm and nuclei (Fig. 1).

### LPA promotes the expression of CXCR4 in ovarian cancer cells in a time- and dose-dependent manner

**Figure 2:**
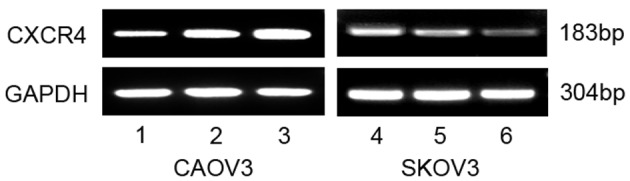
Effects of LPA at different doses on CXCR4 mRNA expression in ovarian cancer cells by RT-PCR. 1, CAOV3 control group; 2, CAOV3 5-μM LPA 24-h group; 3, CAOV3 20-μM LPA 24-h group; 4, SKOV3 20-μM LPA 24-h group; 5, SKOV3 5-μM LPA 24-h group; and 6, SKOV3 control group. RT-PCR, reverse transcritpion polymerase chain reaction; LPA, lysophosphatidic acid; CXCR4, C-X-C motif chemokine receptor 4.

**Figure 3:**
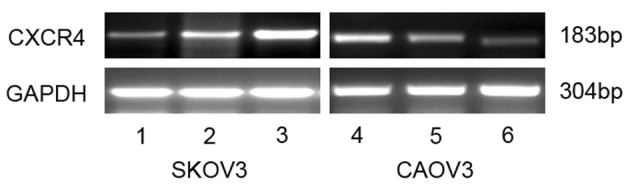
Effects of LPA with different times on CXCR4 mRNA expression in ovarian cancer cells by RT-PCR. 1, SKOV3 control group; 2, SKOV3 20-μM LPA 12-h group; 3, SKOV3 20-μM LPA 24-h group; 4, CAOV3 20-μM LPA 24-h group; 5, CAOV3 20-μM LPA 12-h; and 6, CAOV3 control group. RT-PCR, reverse transcription polymerase chain reaction; LPA, lysophosphatidic acid; CXCR4, C-X-C motif chemokine receptor 4.

Fluid cytology results demonstrated that when the CAOV3 and SKOV3 cells were treated with different LPA concentrations (0, 5 and 20 μM) for 24 h, the CXCR4 expression levels were higher compared with the control. X-mode values of the relative CXCR4 protein level on the CAOV3 and SKOV3 cell membranes increased significantly (P<0.01) compared with the control group, when cells were treated with LPA at a concentration of 20 μM for 24 h. The RT-PCR results showed that LPA increases the mRNA expression of CXCR4 in ovarian cancer cells (Figs. 2 and 3).

### LPA induces ovarian cancer cells to secrete CXCL12 in a time- and dose-dependent manner

**Figure 4:**
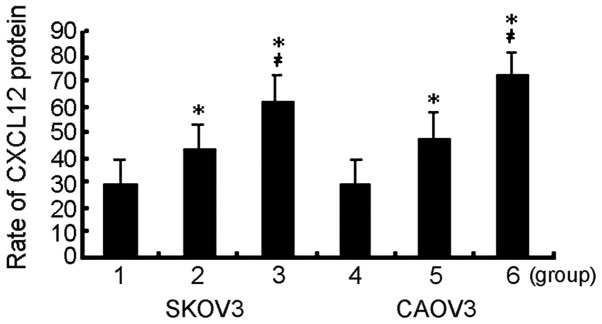
Expression of CXCL12 protein in ovarian cancer cell lines, CAOV3 and SKOV3, in groups treated with different doses of LPA, as determined by ELISA. 1, SKOV3 control group; 2, SKOV3 5-μM LPA 24-h group; 3, SKOV3 20-μM LPA 24-h group; 4, CAOV3 control group; 5, CAOV3 5-μM LPA 24-h group; and 6, CAOV3 20-μM LPA 24-h group. ^*^P<0.05 vs. control group; and ^#^P<0.05 vs. 5-μM LPA 24-h group. LPA, lysophosphatidic acid; CXCL12, C-X-C motif chemokine ligand 12.

**Figure 5:**
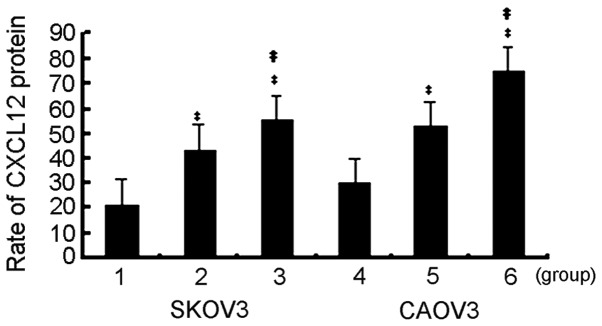
Expression of CXCL12 protein in ovarian cancer cell lines, SKOV3 and CAOV3, in groups treated for different times with LPA, as determined by ELISA. 1, SKOV3 control group; 2, SKOV3 20-μM LPA 12-h group; 3, SKOV3 20-μM LPA 24-h group; 4, CAOV3 control group; 5, CAOV3 20-μM LPA 12-h group; and 6, CAOV3 20-μM LPA 24-h group. ^*^P<0.05 vs. control; and ^#^ P<0.05 vs. 20-μM LPA 12-h group. LPA, lysophosphatidic acid; CXCL12, C-X-C motif chemokine ligand 12.

ELISA results showed that the ovarian cancer cells, CAOV3 and SKOV3, were stimulated by different LPA concentrations (0, 5 and 20 μM) for 24 h, and that the expression of CXCL12 protein in the supernatant significantly increased in a dose-dependent manner, with a significant difference among each group (F=34.871, P=34.871; and F=14.218, P=14.218, respectively). When the cells were treated with 20 μM LPA for the varying times (0, 12 and 24 h), the expression of the CXCL12 protein significantly increased in a time-dependent manner and there was a significant difference among each group (F=13.53, P=0.001; and F=24.54, P=24.54, respectively) (Figs. 4 and 5).

### Effect of the CXCL12-CXCR4 axis regulated by LPA on ovarian cancer proliferation

**Figure 6:**
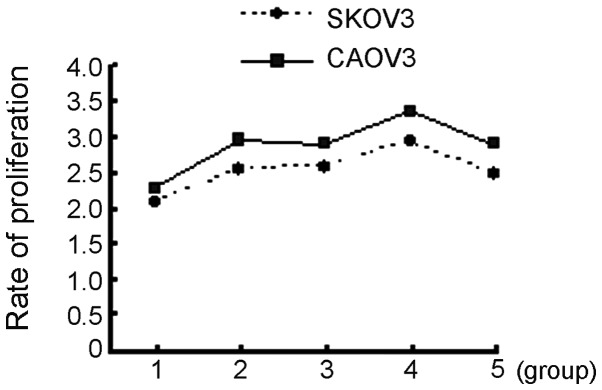
Cell proliferation of CAOV3 and SKOV3 cells in the different groups, as determined by MTT. 1, Control group; 2, 20-μM LPA group; 3, 100-ng/ml CXCL12 group; 4, 20-μM LPA + 100-ng/ml CXCL12 group; and 5, 20-μM LPA + 100-ng/ml CXCL12 + 100-ng/ml PTX group. LPA, lysophosphatidic acid; CXCL12, C-X-C motif chemokine ligand 12; PTX, pertussis toxin.

When using serum-free media, the MTT results showed that CXCL12 (100 ng/ml) could stimulate the proliferation of the ovarian cancer cells, CAOV3 and SKOV3 (P<0.0001 and P<0.0001, respectively), however, when the cells were treated with LPA (20 μM) and CXCL12 (100 ng/ml) simultaneously for 24 h, the proliferation was significantly enhanced compared with the CXCL12 group (P=0.001 and P=0.041, respectively). However, additional treatment with PTX (100 ng/ml), a Gi protein inhibitor, inhibited the enhanced proliferation by LPA and CXCL12 (Fig. 6).

### Effect of the CXCL12-CXCR4 axis regulated by LPA on ovarian cancer cell migration and invision

**Figure 7:**
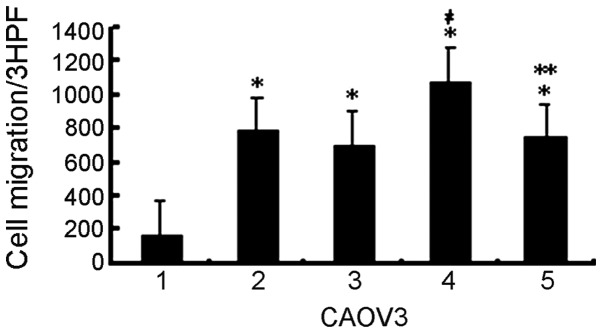
Migration of CAOV3 cells in the different groups, as observed by HE staining. 1, Control group; 2, 20-μM LPA group; 3, 100-ng/ml CXCL12 group; 4, 20-μM LPA + 100-ng/ml CXCL12 group; and 5, 20-μM LPA + 100-ng/ml CXCL12 + 100-ng/ml PTX group. ^*^P<0.01 vs. control group; ^#^ P<0.01 vs. 100-ng/ml CXCL12 group; and ^**^P<0.01 vs. 20-μM LPA + 100-ng/ml CXCL12 group. HE, hematoxylin and eosin; LPA, lysophosphatidic acid; CXCL12, C-X-C motif chemokine ligand 12; PTX, pertussis toxin.

**Figure 8:**
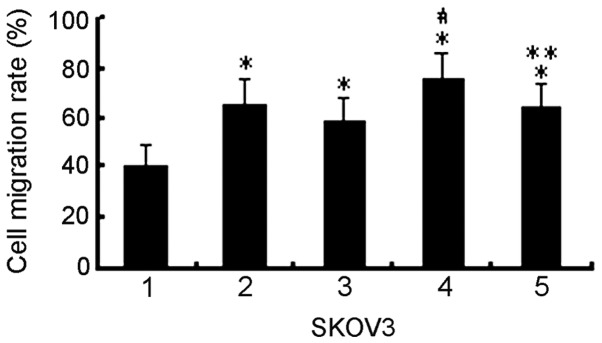
Migration of SKOV3 cells in the different groups, as determined by MTT. 1, Control group; 2, 20-μM LPA group; 3, 100-ng/ml CXCL12 group; 4, 20-μM LPA + 100-ng/ml CXCL12 group; and 5, 20-μM LPA + 100-ng/ml CXCL12 + 100-ng/ml PTX group. ^*^P<0.05 vs. control group; ^#^P<0.01 vs. 100-ng/ml CXCL12 group; and ^**^ P<0.05 vs. 20-μM LPA + 100-ng/ml CXCL12 group. LPA, lysophosphatidic acid; CXCL12, C-X-C motif chemokine ligand 12.

The migration rate of the CAOV3 and SKOV3 cells was detected by HE staining and MTT, respectively. Following treatment with 100 ng/ml CXCL12 for 24 h, the migration rates of the ovarian cancer cells, CAOV3 and SKOV3, were significantly increased compared with the control group (P<0.0001 and P=0.005, respectively). However, the rate of the cell migration of cells stimulated with 100 ng/ml CXCL12 and 20 μM LPA simultaneously was significantly increased compared with the CXCL12 group (P<0.0001 and P<0.0001, respectively). However, additional treatment with PTX (100 ng/ml) inhibited the enhanced migration by LPA and CXCL12 (Figs. 7 and 8).

## Discussion

Ovarian cancer is the most fatal of the gynecological tumors with a 5-year survival rate of only 30%, as patients succumb to peritoneal cavity metastatic lesions in the omentum, gastrointestinal, liver, spleen and other organs, due to unique peritoneal metastasis (14). Thus it is important to study the transfer mechanism and to disrupt the methods of ovarian cancer metastasis, with an aim to improve the survival rate of patients. Invasion and metastasis are complex processes with multiple steps and factors, including the adhesion of β1 integrin, the degradation of the basement membrane mediated by MMP and angiogenesis induced by VEGF (3,10,15). However, ovarian cancer cells can migrate and invade to the human peritoneal mesothelial cells due to the involvement of chemokines and the specific expression of their receptors. So far, the chemokine CXCL12-CXCR4 axis in tumor metastasis has been confirmed in breast cancer (16), non-small cell lung cancer (17) and prostate cancer (18). Tumors with a high expression of CXCR4 possibly metastasize to specific organs that secrete its ligands attracted by CXCL12, resulting in the formation of organ-specific metastasis. The CXCL12-CXCR4 axis can promote tumor growth, angiogenesis and enhance invasion and migration (19).

Our preliminary study results showed a high expression of CXCR4 and CXCL12 in ovarian cancer tissues, but no expression in normal ovarian epithelial cells. The CXCR4 expression was correlated with lymph node metastasis, and the expression intensity of CXCL12 was associated with the volume of ascites (13). A study by Guo *et al* (20) confirmed that ovarian cancer with CXCR4 expression was often accompanied by lymph node metastasis. A recent study demonstrated that all 289 ovarian cancer patients analyzed expressed CXCL12, and the rate of CXCR4 expression was 69%; thus CXCL12 can serve as an independent prognostic indicator for ovarian cancer (15).

LPA can activate growth factors, including VEGF, and play significant roles via special G protein-coupled receptors on the cell surface. As a bioactive molecule, LPA can induce cell proliferation, migration and apoptosis, and often works as a tumor marker. (21,22) A large number of studies have shown that the increasing expression of LPA in ovarian cancer cells may play a key role in the transfer and invasion process, indicating that LPA may promote tumor invasion and metastasis by upregulating the expression of certain genes associated with tumor metastasis (5–9,23). We hypothesized that LPA possibly affects the metastasis of ovarian cancer cells by enhancing chemotaxis, and there may be a certain association between LPA and the CXCL12-CXCR4 axis in the metastasis of tumors.

In the present study, CXCR4 was expressed in the cell membrane and cytoplasm of the ovarian cancer cell lines, SKOV3 and CAOV3. LPA directly upregulated the expression of CXCR4 in a dose- and time-dependent manner. The results of the present study also confirmed that LPA increased the content of CXCL12 in the supernatant of ovarian carcinoma cells and enhanced the proliferation, migration and invasion of the ovarian cancer cells, SKOV3 and CAOV3. Taken together, the results of the present study demonstrated that LPA could upregulate the expression of the CXCL12-CXCR4 axis, which was a novel mechanism for LPA to promote the metastasis and infiltration of ovarian cancer cells.

As a lipid second messenger, LPA acts on target cells mainly through the G protein-coupled receptor signal pathway. The LPA receptor, coupled with ~3 different G proteins, activates the coupling G protein and further activates the signaling pathway mediated by the G protein, resulting in gene expression or protein change. These 3 G proteins are Gi, Gq and G12/13 (24–26). PTX is a Gi protein inhibitor (27). LPA inhibits adenylate cyclase activity through the Gi protein, so as to suppress cyclic adenosine monophosphate (cAMP) formation, as increased cAMP can inhibit cell growth. Therefore, LPA realizes the growth factor function in this manner. The present study assessed the effect of the CXCL12-CXCR4 axis regulated by LPA on ovarian cancer cell proliferation, migration and invasion, and demonstrated that PTX inhibited the regulation of CXCL12 by LPA, and decreased the proliferation, migration and invasion ability of ovarian cancer cells that were enhanced by CXCL12 and LPA. This indicates that LPA regulated the CXCL12-CXCR4 axis in the endothelial differentiation gene/LPA receptor family through the signaling pathway mediated by the Gi protein.

LPA is involved in the mechanism of the pathogenesis and metastasis of ovarian cancer, and can serve as a main target of ovarian cancer treatment. Further study on the mechanism of ovarian cancer metastasis promoted by LPA would be a significant step from basic research to clinical application. The present study confirmed that LPA could upregulate the expression of the CXCL12-CXCR4 axis so as to promote the metastasis of ovarian cancer, but this mechanism requires further investigation. LPA is a small molecule possessing a wide spectrum of biological functions. The specific treatment of ovarian cancer with LPA is an enormous challenge. However, the inhibition of the expression of LPA or the LPA receptor will be a novel molecular therapy for ovarian cancer metastasis.
